# Occurrence of Neuroblastoma among *TP53* p.R337H Carriers

**DOI:** 10.1371/journal.pone.0140356

**Published:** 2015-10-09

**Authors:** Ana Luiza Seidinger, Fernanda Paschoal Fortes, Maria José Mastellaro, Izilda Aparecida Cardinalli, Lilian Girotto Zambaldi, Simone Santos Aguiar, José Andrés Yunes

**Affiliations:** 1 Molecular Biology Laboratory, Boldrini Children’s Center, Campinas, Sao Paulo, Brazil; 2 Pediatric Oncology Department, Boldrini Children’s Center, Campinas, Sao Paulo, Brazil; 3 Pathology Department, Boldrini Children’s Center, Campinas, Sao Paulo, Brazil; 4 Cytogenetics Laboratory, Boldrini Children’s Center, Campinas, Sao Paulo, Brazil; 5 Center for Research in Pediatrics, Faculty of Medical Sciences, State University of Campinas, Campinas, Sao Paulo, Brazil; 6 Medical Genetics Department, Faculty of Medical Sciences, State University of Campinas, Campinas, Sao Paulo, Brazil; Ospedale Pediatrico Bambino Gesù, ITALY

## Abstract

The high incidence of adrenocortical tumors and choroid plexus carcinoma in children from South and Southeastern regions of Brazil is associated with the germline p.R337H mutation of *TP53* gene. The concomitant occurrence of neuroblastoma and adrenocortical tumors in pediatric patients harboring the p.R337H mutation at our institution prompted us to investigate the putative association between p.R337H and pediatric neuroblastoma. Genomic DNA samples from 83 neuroblastoma patients referred to a single institution during the period of 2000–2014 were screened for the p.R337H mutation. Available samples from carriers were investigated for both nuclear p53 accumulation and loss of heterozigosity in tumor. Clinical data were obtained from medical records in order to assess the impact of 337H allele on manifestation of the disease. Seven out 83 neuroblastoma patients (8.4%) were carriers of the *TP53* p.R337H mutation in our cohort. Immunohistochemical analysis of p.R337H-positive tumors revealed nuclear p53 accumulation. Loss of heterozigosity was not found among available samples. The presence of 337H allele was associated with increased proportion of stage I tumors. Our data indicate that in addition to adrenocortical tumors, choroid plexus carcinoma, breast cancer and osteosarcoma, genetic counseling and clinical surveillance should consider neuroblastoma as a potential neoplasia affecting p.R337H carriers.

## Introduction

*TP53* is a tumor suppressor gene involved in the etiology of a variety of tumors [1]. Germline mutations in this gene are usually found in families presenting Li-Fraumeni syndrome (LFS) or Li-Fraumeni-like syndrome (LFLS). These clinical conditions predispose individuals to a wide spectrum of early-onset cancers, including soft tissue and bone sarcomas, central nervous system (CNS) tumors, adrenocortical tumors (ACT), breast cancer and leukemia [2–13].

The germline mutation p.R337H of *TP53* gene has an unusually high prevalence in Brazil, reaching 0.3% of the healthy population from Southern region [14,15]. Although its tumorigenic effect initially appeared to be tissue-specific, being associated with only ACT [16], we and others found evidences indicating its association with a broader spectrum of human malignancies, e.g. breast cancer, choroid plexus carcinoma, osteosarcoma, phyllodes tumors of the breast and LFLS families [17–24].

In a preliminary study at our institution we identified two carriers of p.R337H mutation presenting concomitant ACT and neuroblastoma (NB) (data presented hereafter), indicating a putative role for p.R337H on NB tumorigenesis. The surveillance program developed in Southern Brazil for the early diagnosis of cancer among children carriers of the p.R337H, reported, as expected, occurrence of ACT and choroid plexus carcinoma, and less frequently glioblastoma multiforme, Burkitt lymphoma and neuroblastoma [15].

Neuroblastoma is an embryonal tumor of the sympathetic nervous system, derived from primordial neural crest cells. Together with ganglioneuroblastoma and ganglioneuroma, neuroblastoma constitutes the group of neuroblastic tumors. NB is the most immature, and malignant form of neuroblastic tumor and it arises almost exclusively in infants and young children. The most frequent identified primary sites are adrenal medulla and paravertebral sympathetic ganglia. NB is a remarkably heterogeneous neoplasia, presenting spontaneous regression and differentiation in some infants, while children with high-risk disease often present resistance to therapy [25].

NB is not commonly associated with *TP53* mutations [26]. Recently, a single nucleotide polymorphism (SNP) that maps to 3’ UTR of *TP53* (rs78378222) was found to be associated with neuroblastoma susceptibility [27]. This germline variant impairs proper termination and polyadenylation of *TP53* transcripts and besides NB, it was also found to confer susceptibility to cutaneous basal cell carcinoma, prostate cancer, glioma and colorectal adenomas [28].

Although the role of *TP53* on neuroblastoma tumorigenesis is still under debate, our preliminary findings prompted us to investigate the association between the highly prevalent p.R337H mutation and pediatric neuroblastoma. In addition, we investigated the presence of SNP rs78378222 in our cohort and the impact of 337H allele on clinical manifestation and prognosis of this disease.

## Material and Methods

### Patients

The subjects included in the current study comprised pediatric patients diagnosed and treated for neuroblastoma at a single institution located in Campinas, São Paulo, Brazil, during the year 2000 through July 2014. During this period, 178 patients were diagnosed with neuroblastoma and classified according to International Neuroblastoma Staging System (INSS) and Shimada criteria [29,30]. The 83 patients enrolled in this study were selected only on the basis of availability of samples for the p.R337H mutation investigation. The samples studied included both peripheral blood mononuclear cells (MNCs) and/or tumor samples. The cancer family history, *MYCN* status, demographic and clinical data were obtained from patients’ medical records.

### Ethics Statement

This study was approved by the Ethical Research Committee of the Faculty of Medical Sciences at the State University of Campinas (approval number 1121/2008), which waived the signature of informed consent because the work was conducted using retrospective samples from tumor bank.

### Screening for p.R337H mutation

Genomic DNA was isolated from peripheral blood or tumor samples by using a standard phenol:chlorophorm extraction method [31] followed by a PCR to amplify the exon 10 of *TP53* gene. The PCR product was digested with the restriction enzyme *Hha*I (Fermentas Inc.), which yields 2 fragments (293 bp and 154 bp) in the wild-type amplicon but only 1 fragment when the p.R337H mutation is present [16]. The presence of p.R337H mutation was confirmed by Sanger sequencing by using the BigDye Terminator Cycle Sequencing Ready Reaction Kit (Applied Biosystems) in an ABI PRISM 310 automated sequencer (Applied Biosystems).

### Loss of heterozygosity analysis

Paired DNA samples from germline and tumor tissue were investigated for loss of heterozigosity (LOH). p.R337H allelic discrimination was achieved through custom made TaqMan SNP genotyping. Assay standardization and validation in more than 30,000 samples will be presented elsewhere (Caminha IP et al. in preparation). Briefly, genomic DNA was subjected to qPCR using primers that flank the region of the p.R337H mutation: 5’-CCTCCTCTGTTGCTGCAGATC-3’ and 5’-CCTCATTCAGATCTCTCGGAAC-3’ in conjunction with two MGB probes: 5’-VIC-CGTGAGC**G**CTTCGAG-3’ and 5’-FAM-CGTGAGC**A**CTTCGAG-3’, that bind to the wild-type and mutant allele, respectively. The reaction consisted of 6μL of 2x TaqMan® Universal PCR Master Mix (Life Technologies), 0,2 μM of each probe (Life Technologies), 0,9 μM of each primer in a final volume of 12 μL. The cycling conditions consisted of 95°C for 10 minutes, followed by 40 repetitions of a two-step cycle (15 seconds at 95°C and 1 minute at 60°C) in a Step One Real-Time PCR System (Life Technologies). Since a probe for each allele was included in the reaction, heterozygous samples show signal amplification for both probes. Homozygous samples or samples that have lost the heterozygosity show signal amplification from only one probe.

### Screening for mutations within the *TP53* DNA-binding domain

Tumor samples from patients with p.R337H were screened for other possible mutations in exons 5 to 9 of *TP53* gene according to IARC protocol, available in http://p53.iarc.fr/ProtocolsAndTools.aspx. PCR products were sequenced based on Sanger method by using the BigDye Terminator Cycle Sequencing Ready Reaction Kit (Applied Biosystems) in an ABI PRISM 310 automated sequencer (Applied Biosystems). Sequences obtained were compared to NCBI reference sequence NG_017013.2. Suspected mutations were confirmed by amplifying a new PCR product from the same DNA sample, followed by a new sequencing reaction.

### Genotyping for SNP rs78378222

This hypomorphic allele is characterized by an A to C transversion at the 3’UTR of *TP53* (NM_000546.5:c.*1175A>C). In order to genotype the patients included in our cohort, we amplified the correspondent region with primers rs78378222F: 5’- GTAAAACGACGGCCAGTGGGTCAACATCTTTTACATTC-3’ and rs78378222R: 5’- TAATACGACTCACTATAGGGCCAGCACCTCCTCACTCAC-3’ by using standard PCR conditions. These primers were tagged with M13 and T7 universal primers (underlined sequences), which were used for sequencing in an ABI PRISM 310 automated sequencer (Applied Biosystems), by using the BigDye Terminator Cycle Sequencing Ready Reaction Kit (Applied Biosystems). The sequences were compared to the reference sequence of *TP53* (NG_017013.2).

### Immunohistochemistry

After dewaxing and rehydration, 5-μm-thick tumor sections were treated with H_2_O_2_ to reduce endogenous peroxidase activity and then underwent wet heat-mediated antigen retrieval with TRIS-ethylene diamine tetra acetic acid, pH 8.9. Sections were incubated with mouse monoclonal anti-p53 antibody (clone DO-7; Dako A/S). This antibody is specific for an N-terminal epitope and reacts with both wild-type and mutant human p53 proteins. Ten NB tumors negative for p.R337H were stained in parallel as negative controls. Colon adenocarcinoma sections, known to stain positively for p53, were stained in parallel as positive controls. Results were observed by using the standard avidin-biotin complex method with the Dako LSAB System horseradish peroxidase kit in a Nikon Eclipse E200 microscope (Nikon Instruments).

### Statistical Analysis

For statistical purposes, the primary site of disease was categorized as adrenal medulla or non-adrenal. The association between p.R337H presence and gender, age at diagnosis, tumor stage at diagnosis, primary site of disease, *MYCN* amplification and outcome was assessed by the Mann Whitney test, Chi-square test for trend or Fisher Exact test. Statistical analyses were performed by using Graphpad Prism 5.0 (GraphPad Software Inc., San Diego, CA).

## Results

### *TP53* findings among NB patients

**Fig 1:**
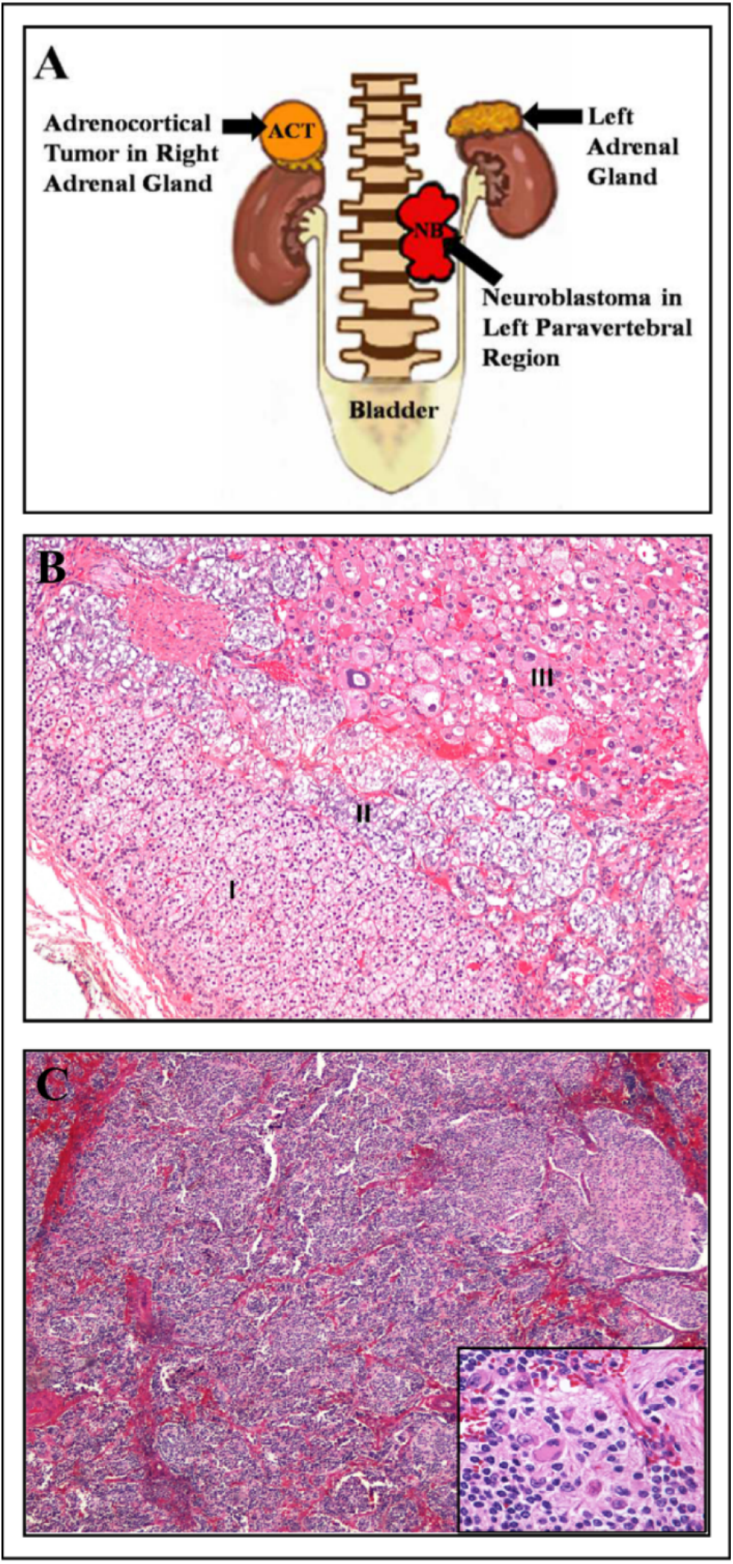
Morphological and histological characterization of tumors from patient #1, presenting concomitant NB and ACT. A) Schematic representation based on patient #1 image files demonstrating adrenocortical tumor in right adrenal gland and the neuroblastoma in left paravertebral region. B) Right adrenocortical tumor from patient #1 measuring 2,0 x 1,8 x 0,8 cm. The microscopic examination revealed in I) cortical region of non-tumoral adrenal; II) medullary region of non-tumoral adrenal; III) adrenocortical tumor characterized by atypical large pleomorphic cells with acidophilic cytoplasm, presenting Fuhrman nuclear atypia grade 3 (H&E 100x magnification). C) Histological examination of left paravertebral tumor from patient #1. The neuroblastoma presented dimensions of 4,3 x 3,2 x 1,5 cm and was characterized by small round primitive cell clusters (H&E staining, 40x magnification). In detail, neuroblasts at different maturation stages embedded in neurofibrillary stroma (H&E, 400x magnification).

**Fig 2:**
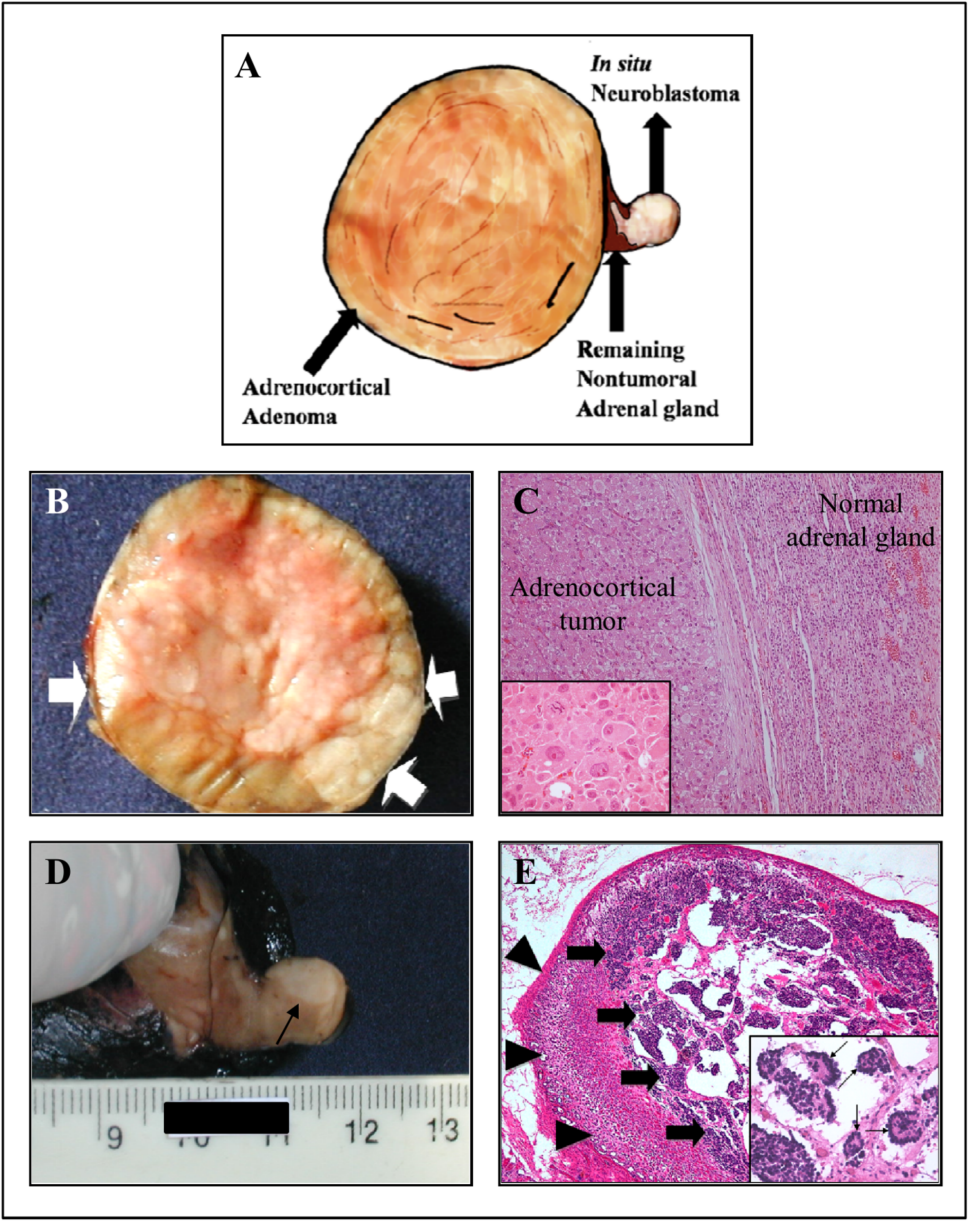
Morphological and histological characterization of tumors from patient #2, presenting concomitant NB and ACT. A) Schematic representation based on patient #2 macroscopic findings shows the right adrenal gland presenting concomitant occurrence of adrenocortical adenoma and *in situ* neuroblastoma. B) The macroscopic examination of tumor demonstrates a solid and well-circumscribed adrenocortical tumor, completely surrounded by a thin fibrous capsule (arrow). The cut surface shows a yellowish, shiny, and homogeneous neoplasm with slightly lobular architecture. C) The left half of the picture shows the adrenocortical tumor surrounded by a thin and fibrous capsule (HE, 100x). Insert a detail demonstrating tumor cells of large size, with huge and eosinophilic cytoplasm, large nuclei, with central and conspicuous nucleoli. (HE, 400x). D) *In situ* neuroblastoma can be identified as a slightly pale region indicated by an arrow. E) Microscopic analysis from the pale region of remainder adrenal gland shows well-preserved cortical parenchyma cells (arrowheads). The inner medullary zone is heavily infiltrated by *in situ* neuroblastoma (arrows) (HE, 40x). Insert, a detail showing tumor composed of small round blue cells with scant cytoplasm, and dark, hyperchromatic nuclei with frequent formation of Homer-Wright rosettes (thin arrows) (HE, 100x).

Patients diagnosed with adrenocortical tumor at our institution are invited to do the p.R337H testing due the strong association of this mutation with ACT at our geographic region [15–17,19,22]. Two p.R337H heterozygous ACT patients were found to present synchronous neuroblastoma (Figs 1 and 2). A complete description of clinical characteristics of these two patients was summarized in S1 Table. This finding led us to hypothesize that p.R337H could be actively associated to NB tumorigenesis.

We investigated therefore the presence of p.R337H in an additional group of 81 pediatric patients diagnosed exclusively with neuroblastoma. In this group, we identified other 5 carriers of this mutation. The presence of p.R337H mutation in patients with NB was confirmed by using at least two methodologies. The 7 patients harboring the p.R337H mutation (2 diagnosed with ACT plus NB and 5 with solely NB) accounted for 8.4% of tested patients. None of 77 tested patients from our cohort (p.R337H positive n = 7 / p.R337H negative n = 70) was found to carry the hypomorphic allele (C) for SNP rs78378222.

*TP53* mutations in NB may arise as a consequence of tumor progression or be induced by chemotherapy [32,33]. In our cohort, only two out seven p.R337H positive tumors were subjected to chemotherapy before mutation genotyping. Nevertheless, the mutation was also detected in blood samples from these two patients, confirming that the mutation was germline and was not acquired in consequence of a cytotoxic exposure (Table 1). Direct sequencing of exons 5–9 of *TP53* gene revealed no additional mutation on p.R337H positive tumors (Table 1).

**Table 1:** Characteristics of p.R337H positive patients.

Patient	Diagnosis	Tissue screened for p.R337H	Codon 337 status	DBD mutation	IHC p53 tumor	Cytotoxic therapy before DNA analysis of tumor?
1	Neuroblastoma IV + ACT	Blood	Arg/His	No	NB–+	Yes
		Tumor (NB)	Arg/His		ACT–++++	
2	Neuroblastoma *in situ* adrenal + ACT	Blood	Arg/His	NA	NB–NA	NA
					ACT–++	
3	Neuroblastoma III	Tumor	Arg/His	No	+++	No
4	Neuroblastoma III	Tumor	His/(His?)^a^	No	++	No
5	Neuroblastoma IV	Blood	Arg/His	No	+++	Yes
		Tumor	Arg/His			
6	Neuroblastoma I	Tumor	Arg/His	No	++++	No
7	Neuroblastoma III	Blood	Arg/His	No	++++	No
		Tumor	Arg/His			

NB–Neuroblastoma; ACT–adrenocortical tumor; NA–material not available for analysis; IHC–immunohistochemistry; DBD–DNA-binding domain. Immunohistochemistry was scored as 0 (no cells positive), + (up to 25% of cells positive), ++ (26% to 50% of cells positive), +++ (51% to 75% of cells positive), or ++++ (>75% of cells positive).

^a^ Only the mutant allele was found in this tumor. It was not possible to distinguish between homozygosity or loss of heterozygosity with retention of the mutated allele in the tumor.

**Fig 3:**
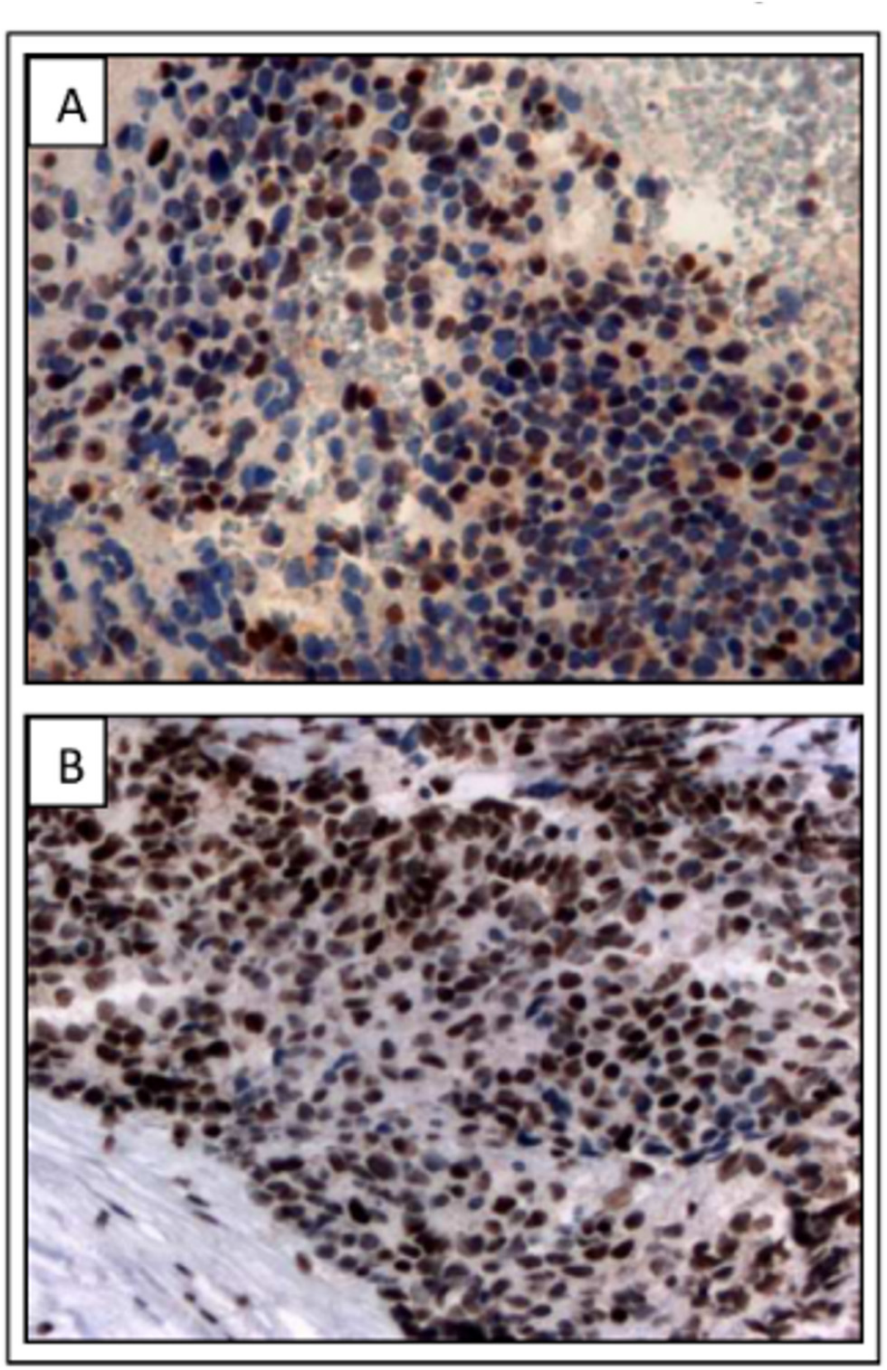
Immunohistochemical p53 staining on p.R337H positive NB tumors. A, B) Representative images of nuclear p53 accumulation on neuroblastoma cells from patients 3 and 7, respectively (100x magnification).

Although LOH was not found among the p.R337H NB patients available for this analysis, immunohistochemistry against p53 revealed nuclear protein accumulation on p.R337H positive tumors, reinforcing the hypothesis of p53 inactivation on NB cells (Table 1; Fig 3). The median expression pattern of the 10 negative p.R337H NB tumors, used as a reference for this assay, was 15% of NB cells presenting immunopositivity for p53.

### Demographical and clinical data of NB patients

Table 2 summarizes the clinical and demographical data for NB patients included in the present study, according to p.R337H status. No significant association was observed between p.R337H presence and gender, age at diagnosis, primary site of disease, *MYCN* amplification and outcome. p.R337H was statistically associated with increased proportion of stage I tumors. These findings should be considered with caution, due to the small number of p.R337H patients analyzed.

**Table 2:** Clinico-biological data of neuroblastoma patients according to p.R337H status.

		Positive p.R337H n (%)	Negative p.R337H n (%)	*p* value
Overall number		7	76	-
**Gender**	Male	3 (43%)	40 (53%)	0.7 ^F^
	Female	4 (57%)	36 (47%)	
**Age at diagnosis**	Median	27 months	22 months	0.8 ^M^
**Stage at diagnosis**	I	2 (28.5%)	4 (5.2%)	0.04^X^
	IIA/IIB	0	5 (6.6%)	
	III	3 (43%)	23 (30.2%)	
	IV	2 (28.5%)	42 (55.3%)	
	IVS	0	2 (2.7%)	
**Site of primary disease**	Adrenal	3 (43%)	36 (47%)	1 ^a^ ^,^ ^F^
	Non-Adrenal	4 (57%)	40 (53%)	
***MYCN* status**	Normal	3 (43%)	37 (49%)	1 ^b^ ^,^ ^F^
	Amplified	3 (43%)	33 (43%)	
	Unknown	1 (14%)	6 (8%)	
**Outcome**	Alive	5 (71%)	32 (42%)	0.28^c^ ^,^ ^F^
	Death	2^d^ (29%)	42 (55%)	
	Lost follow up	0	2 (3%)	
	Median time follow up	55 mo	28 mo	

^F^ Fisher Exact test

^M^ Mann Whitney test

^X^ Chi-square test for trend

^a^ For statistical purposes, the primary site of disease was categorized as adrenal or non-adrenal.

^b^ The statistical analysis included only patients with known *MYCN* status.

^c^ The statistical analysis did not include patients who were lost of follow up.

^d^ Patient 4 and 7 in Table 1.

The cancer family history was investigated at the time of diagnosis. The interview was conducted by an oncologist and data obtained were based only on patient’s report, with no further confirmation by pathology report. Among p.R337H negative patients with available information (n = 61), 39 patients mentioned sporadic cases in family members, while no family met the criteria for LFS or LFLS (Table 3). Among the p.R337H positive patients (n = 7), 4 reported sporadic cases in family members and one met the criteria for LFLS (Birch) associated with the parental side segregating the p.R337H mutation (Table 3). Among the negative patients, the most frequent sites reported were uterus (n = 6), leukemia (n = 6), skin (n = 5), breast (n = 4) and prostate (n = 4). Among the positive individuals, the most frequent sites reported were breast (n = 2), esophagus (n = 2) and lung (n = 2).

**Table 3:** Cancer family history of the 83 neuroblastoma patients according to p.R337H status.

	Positive p.R337H (n = 7)	Negative p.R337H (n = 76)
No information available	0	15
**Absence of cancer history**	2 (29%)	22 (36%)
**Sporadic cases** ^**a**^	4 (57%)	39 (64%)
**LFS/LFLS**	1 (14%)	0 (0%)

^a^ Presence of at least one member of the family with cancer, without criteria for a cancer predisposition syndrome though.

## Discussion

Since its description in 2001 by Ribeiro and colleagues [16], continuous efforts have been devoted to better understand how the p.R337H mutation contributes to carcinogenesis. Although initially controversial, its association with a broad spectrum of tumors is now well accepted. Strongly associated with ACT and CPC, the 337H allele is thought to be responsible for the high incidence of these tumors in the Southern and Southeastern regions of Brazil [16,19,20,22]. Besides ACT and CPC, osteosarcoma and breast cancer, including phyllodes tumors of the breast, are also associated with p.R337H, in a lesser extent though [18,19,21,23,24]. In the present work, we describe the identification of p.R337H carriers among neuroblastoma pediatric patients. Seven out of 83 patients tested (8.4%) were carriers of 337H allele. This frequency is about 28 to 42 times higher than the estimated 0.2 to 0.3% frequency for p.R337H in people not selected by cancer diagnosis from our region [14,15], suggesting that carriers of the p.R337H are at increased risk of developing NB than the general population. Although cancer in general does not arise from a single gene defect, populations in which p.R337H was identified should consider neuroblastoma as a potential neoplasia affecting carriers. In accordance with our findings, Custódio and colleagues have also identified a neuroblastoma patient in a surveillance program for p.R337H carriers in Southern Brazil (Table 4) [15].

**Table 4:** Literature review on *TP53* polymorphisms in neuroblastoma.

*TP53* polymorphisms in neuroblastoma							
Study design	Polymorphism	Location	Nucleotide change	Amino acid change	Minor allele frequency (MAF)^a^	MAF among NB	Reference
Cohort of 41 NB patients	rs1042522	exon 4 (codon 72)	C**C**C—C**G**C	Pro to Arg	0.457	0.1375	[45]
Cohort of 286 NB patients	rs1042522	exon 4 (codon 72)	C**C**C—C**G**C	Pro to Arg	0.457	0.20	[46]
Cohort of 2804 NB patients	rs35850753	5’ UTR Δ133p53	**G**—**A**	-	0.0056	0.036	[27]
	rs78378222	3’ UTR	**A**—**C**	-	0.0026	0.027	
Cohort of 2101 NB patients	rs8079544	intron 1	**G**—**A**	-	0.0783	0.068	
Cohort of 1809 NB patients	rs1042522	exon 4 (codon 72)	C**C**C—C**G**C	Pro to Arg	0.457	0.25	

^a^ The minor allele frequency for a given SNP is based on its frequency in a default global population. The current default global population is 1000 Genome phase 1 genotype data from 1094 worldwide individuals, released in the May 2011 dataset.

Nuclear p53 accumulation on NB cells suggests p53 inactivation in p.R337H-positive tumors (Fig 3). Paired analysis of germline and tumor tissues revealed no LOH in available cases (Table 1). LOH with retention of the mutated allele was identified in virtually all cases of ACT and CPC associated with p.R337H [19]. On the other hand, the mechanism of breast carcinogenesis associated with p.R337H mutation appears to be not related to the classical two-hit model involving tumor suppressor genes, since LOH at the mutation locus is not common in these cases [24]. It is important to note that p.R337H is a dominant negative mutation that affects the oligomerization domain of p53, so it can interfere with normal function of wild-type allele through the impaired tetramer conformation of the protein [34]. Moreover, different mechanisms of allele inactivation, as promoter methylation and *cis*-acting elements, may also play an important role on allelic imbalance, thus rendering LOH not the exclusive mechanism responsible for reducing expression of the wild-type allele [35–37].

Neuroblastoma is a very heterogeneous malignancy that affects almost exclusively infants and young children. The clinical behavior of NB ranges from spontaneous regression to aggressive tumors that do not respond to current therapies [29]. The presence of p.R337H mutation was statistically associated with increased proportion of stage I tumors. However, due the small number of patients analyzed, this should be taken as a preliminary finding.

Although we had no access to the majority of pathology reports, data obtained on cancer family history of p.R337H positive NB patients showed that only one out of 7 families presented features consistent with LFS/LFLS (Table 3). This finding is in accordance with the broad phenotypic variation observed among families with p.R337H, i.e., a large proportion of families without any history of cancer while some families presenting with clear LFS/LFLS [15–18,22]. This phenotypic variation highlights the importance of penetrance modifying factors, such as low-penetrant mutations and polymorphisms.

Recently, a polymorphism that maps to 3’ UTR of *TP53* (rs78378222) was found to be associated with neuroblastoma susceptibility (Table 4) [27]. None of the NB patients included in our cohort was found to carry the hypomorphic allele at this locus. From our extensive literature revision on *TP53* polymorphisms studied in the context of NB, we found rs1042522 as the most commonly SNP studied among NB patients (Table 4). This polymorphism results in either an arginine (R) or proline (P) at codon 72 (R72P) and although it has been extensively studied, its clinical significance is still unclear according to NCBI SNP database (available in http://www.ncbi.nlm.nih.gov/snp/?term=rs1042522). Interestingly, the allele that codifies for an arginine was consistently overrepresented among neuroblastoma patients (Table 4). Whether R72 is a risk modifying factor for NB remains to be determined.

To our knowledge, 34 patients with neuroblastic tumors harboring *TP53* mutations have been described until now (summarized in Table 5). Considering the cases with somatic alterations, a significant proportion of mutations may have arisen as a consequence of tumor progression or induced by chemotherapy. With respect to 18 patients with germline mutations, we found that p.R337H is the most common inherited *TP53* mutation associated with NB (n = 7). It is intriguing that among the other 11 germline cases described, four (36%) had mutations at codon 248 [38–41]. Noteworthy, one of them presented concomitant NB and ACT and another patient presented ganglioneuroblastoma and ACT [39,41]. Whether neuroblastic tissue present a marked susceptibility to alterations at codon 248 of p53 remains to be investigated. Tissue-specificity of *TP53* mutations has been considerably discussed. Missense *TP53* mutations located in the DNA-binding loop that contact the minor groove of DNA were associated with brain tumors, whereas mutations in the non DNA-binding loops, β-sheets and oligomerization domain were associated with adrenocortical tumors [42]. Mutations affecting *TP53* splicing sites were strongly associated with Wilm’s tumor, while null mutations were not associated with a specific type of tumor, but were associated with early onset tumors, in particular brain tumors [26,42]. Apparently, mutations affecting different domains of the protein may exert different impact on protein function or protein-protein interactions, culminating in different tissue-susceptibility to cancer.

**Table 5:** Literature review on *TP53* mutations in association with neuroblastoma.

NB studies						
Study design	*TP53* region screened	*TP53* mutant / total	Mutation	Germline / Somatic	Diagnosis	Reference
This study	codon 337	7/83	R337H	Germline	Neuroblastoma^a^	-
Case report	Whole exome	1	R248Q	Germline mosaicism and homozygous at tumors	Benign myofibroblastic proliferation – 9 mo; Sarcoma NOS – 11 mo; Neuroblastoma – 1y 4 mo	[38]
Cohort of NB patients	exons 2–11	0/40	-	-	-	[47]
Cohort of NB patients	exons 2–11	2/86	C135Y	Acquired in relapsed tumor	Neuroblastoma	[32]
			G204V	Acquired in metastatic tumor	Neuroblastoma	
Cohort of NB patients	exons 5–9	0/48	-	-	-	[48]
Cohort of NB patients	exons 2–11	0/38	-	-	-	[49]
Cohort of NB patients	exons 5–9	2/20	V172V	No germline tissue available for analysis	Neuroblastoma	[50]
			D259Y	Somatic	Neuroblastoma	
Cohort of NB patients	exons 5–8	0/29	-	-	-	[51]
Case report	exons 5–8	1	C277F	Acquired after chemotherapy	Neuroblastoma – 3y	[33]
Case report	exons 2–11	1	R248W	Germline	ACT – 10 mo and neuroblastoma – 10 mo	[39]
	Not specified	1	Codon 248	Not specified	Neuroblastoma	
Case report	Not specified	1	R248W	Germline	Ganglioneuroblastoma – 1y 6 mo; ACT – 1 y 6 mo; Turner syndrome (45,X)	[41]
Cohort of NB patients	Whole exome	2/240	D281N	Somatic	Neuroblastoma – 5y	[52]
			P219S	Germline	Neuroblastoma – 3y 6 mo	
Cohort of NB patients	exons 5–8	0/30	-	-	-	[53]
Cohort of NB patients	exons 5–8	0/44	-	-	-	[54]
Cohort of NB patients	exons 4–9	6/41	F270L	Acquired after chemotherapy and present at relapse	Neuroblastoma – 1y 7 mo	[45]
			V157G	Acquired after chemotherapy	Neuroblastoma – 7 mo	
			D259Y	At relapse	Neuroblastoma – 2y 3 mo	
			D259Y	Acquired after chemotherapy	Neuroblastoma – 5y 9 mo	
			V203M	At diagnosis	Neuroblastoma – 15y	
			C238Y	Acquired after chemotherapy	Neuroblastoma – 16y	
Cohort of NB patients	exons 4–8	3/40	R273L	Not specified	Neuroblastoma – 12y	[55]
			R283C	Not specified	Neuroblastoma – 1y 10 mo	
			R283C	Not specified	Neuroblastoma – 3 mo	
Case report	Not specified	1	I162F	Germline	Neuroblastoma – 8 mo; ACT – 8 mo	[56]
***TP53* / LFS studies**						
**Study design**	***TP53* region screened**	**NB / total**	**Mutation**	**Germline / Somatic**	**Diagnosis**	**Reference**
Cohort of R337H(+) newborn carriers	codon 337	1/461^b^	R337H	Germline	Neuroblastoma	[15]
Cohort of LFS individuals	All exons, splice junctions and promoter region	3/148^c^	Not specified	Germline	Neuroblastoma	[26]
Families with osteosarcoma	Not specified	1/17 families ^d^	R273H	Germline	Neuroblastoma – 1y	[57]
Cohort of patients with two primary malignant neoplasms and no LFS	exons 5–9	1/59	R248W	Germline	Neuroblastoma – 1 y; Breast carcinoma – 32 y	[40]

NB–neuroblastoma; LFS–Li-Fraumeni Syndrome

^a^ in two cases NB were concomitant with ACT

^b^ of these 461 carriers, 11 developed adrenocortical tumors, 2 choroid plexus tumors, 1 glioblastoma multiforme, 1 Burkitt lymphoma and 1 neuroblastoma

^c^ All 148 cancer cases were diagnosed in carriers of *TP53* germline mutations

^d^ There is no information on the total number of individuals (carriers) analyzed.

From this perspective, it is well accepted that p.R337H predisposes carriers to a broad spectrum of cancer. The spectrum of tumors found in p.R337H families may, eventually, overlap that found in LFS or LFLS [17,18,20]. However, in the majority of cases, this spectrum is not identical [15,16,43,44]. Therefore, the clinical criteria for LFS and LFLS are not suitable for suspecting p.R337H. The challenge in identify individuals carrying the p.R337H mutation and, therefore, family members at high risk of developing cancer, has motivated several efforts to define the exact spectrum of tumors associated to this mutation. The present work shows that in addition to ACT, CPC, breast cancer and osteosarcoma, genetic counseling and clinical surveillance should consider neuroblastoma as a potential disease affecting p.R337H carriers.

## Supporting Information

S1 Table Clinical data of patients diagnosed with synchronous ACT and NB. (DOC)
