# Supplementary material for: Occurrence of Neuroblastoma among TP53 p.R337H Carriers
Source: PLoS One. 2015 Oct 9;10(10):e0140356. doi: 10.1371/journal.pone.0140356 (PMC4599822; doi:10.1371/journal.pone.0140356)
Supplement: S1 Table — (DOC) [file pone.0140356.s001.doc]

Table S1. Clinical data of patients diagnosed with synchronous ACT and NB.

|  | **Patient 1** | **Patient 2** |
| --- | --- | --- |
| Gender | Female | Male |
| Born within the Brazilian p.R337H geographic cluster? | Yes | Yes |
| Age at diagnosis | 24 months | 16 months |
| Year of diagnosis | 2003 | 2001 |
| **ACT** |  |  |
| Symptoms | Virilization | Virilization |
| Primary site | Right adrenal | Right adrenal |
| Stage at diagnosis | I | I |
| Treatment | Surgery + Chemotherapy^a^ | Surgery |
| Outcome | 11 yrs free of disease | 13 yrs free of disease |
| **NB** |  |  |
| Symptoms | Leg and abdominal pain, fever, pallor and proptosis | Asymptomatic |
| Primary site | Retroperitoneum | Right adrenal |
| Stage at diagnosis | IV | I (*in situ* NB) |
| Metastasis | Bone marrow, bones | Absent |
| Treatment | Surgery + Chemotherapy | Surgery |
| Outcome | 9 yrs free of disease | 13 yrs free of disease |

ACT – adrenocortical tumor; NB – neuroblastoma; ^a^ Chemotherapy was administered for NB treatment.
